# HPV status and HPV16 viral load in anal cancer and its association with clinical outcome

**DOI:** 10.1002/cam4.4771

**Published:** 2022-07-04

**Authors:** Daniel Guerendiain, Raluca Grigorescu, Anna Kirk, Andrew Stevenson, Matthew T. G. Holden, Jiafeng Pan, Kim Kavanagh, Sheila V. Graham, Kate Cuschieri

**Affiliations:** ^1^ Scottish HPV Reference Laboratory NHS Lothian Edinburgh UK; ^2^ School of Medicine University of St Andrews St Andrews UK; ^3^ Pathology NHS Lothian Edinburgh UK; ^4^ Centre for Virus Research, Institute of Infection Immunity and Inflammation, College of Medical Veterinary and Life Sciences University of Glasgow Glasgow UK; ^5^ Department of Mathematics and Statistics University of Strathclyde Glasgow UK

**Keywords:** anal cancer, clinical outcome, viral load

## Abstract

**Background:**

The incidence of anal cancer is increasing globally. Evidence‐based improvement in early detection and management of this morbid cancer is thus required. In other cancers associated with Human Papillomavirus (HPV), viral status and dynamics, including viral load (VL) has been shown to influence clinical outcome. Our aim was to determine the influence of HPV status and HPV16 VL on the clinical outcomes of anal cancer patients.

**Methods:**

A total of 185 anal cancer lesions were genotyped for HPV. Of the HPV16 positive component, VL was determined using a digital droplet PCR assay. The association of qualitative HPV status and VL (low (<12.3), medium (12.3–57) and high (>57 copies/cell)) on overall survival and hazard of death was assessed.

**Results:**

Of the 185 cases, 164 (88.6%) samples were HPV positive. HPV16 was detected in 154/185 samples (83.2%). HPV positive status was associated with improved overall survival in the univariate analysis [hazard ratio (HR) of 0.44, 0.23–0.82, *p* = 0.01]. When adjusted by age, sex, stage and response to treatment, the association of positive HPV status with improved survival remained (HR 0.24 [0.11–0.55] *p* < 0.001). High VL was associated with improved overall survival in the univariate analysis with a HR of 0.28 (0.11–0.71, *p* = 0.007). When adjusted only by age and sex, high VL was associated with better overall survival (HR 0.27, 0.11–0.68 *p* = 0.006).

**Conclusions:**

HPV status appears to be independently associated with improved outcomes in anal cancer patients. Moreover, HPV viral load quantification may be informative for further risk stratification and warrants further investigation.

## Graphical abstract

While human papillomavirus (HPV) status has been linked to clinical outcome in oropharyngeal cancer, there are sparse data on how viral presence and dynamics affect the trajectory of anal cancer. This work suggests HPV positive status is independently associated with improved outcomes in anal cancer patients and that viral load measurement shows potential for further risk stratification. Given the rise in anal cancer, identification of biomarkers that can delineate discrete prognostic groups are welcome.

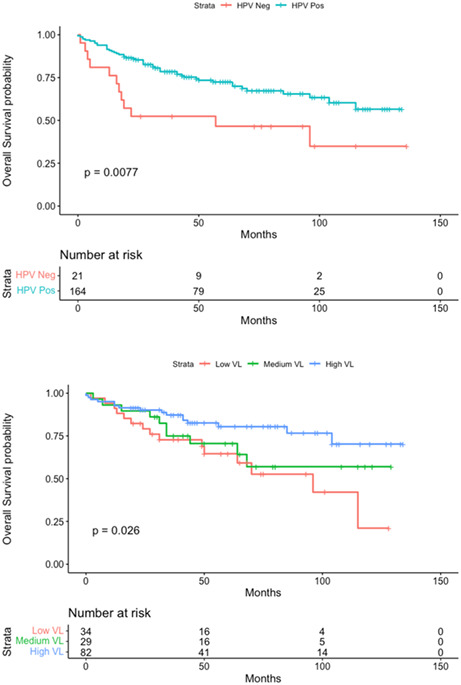

## 1 INTRODUCTION

Anal cancer is one of the six cancers shown to have a human papillomavirus (HPV) aetiology with approximately 90% of anal cancers being HPV‐driven.^1^, ^2^ Like oropharyngeal cancer, there appears to be a clear dominant HPV type driving the lesion. In the meta‐analysis study carried out by De Sanjosè et al. 2019, HPV16 was present in 80.7% of anal cancers.^3^

As with other HPV‐driven cancers, anal cancer incidence is increasing worldwide, including in the USA and Europe.^4^, ^5^, ^6^, ^7^ Scottish European age‐standardised rate (EASR) (per 100,000 person‐years at risk) data align with these increases, rising from 1.5 in 1995 to 2.6 in 2017. For men, incidence increased from 1.6 in 1995 to 2.1 in 2017 while in females, incidence increased from 1.2 in 1995 to 3 in 2017.^8^ The rest of the UK has also experienced an increase for both sexes from 1.5 in 1993 to 1.7 in 2017 in males and 1.3 to 3.0 in females over the same period.^6^

Several factors have been linked to an increased risk of anal cancer including age (higher proportion of cases occur in people after 50 years of age), number of sexual partners, history of receptive anal sexual intercourse, smoking and immune capacity.^9^, ^10^ Women who have or had high‐grade or worse cervical lesions (CIN2/3+) and/or vulvar high‐grade lesions also have a higher risk of anal lesions and cancer.^11^, ^12^, ^13^ Moreover, incidence of anal cancer is significantly higher in people living with HIV (PLWH).^14^, ^15^, ^16^

The morbidity associated with anal lesion and cancer treatments can be very high. Treatments include chemoradiotherapy (CRT) or resection of the affected tissue, depending on the area affected (anal margin cancers are treated in a slightly different way from anal canal tumours).^17^, ^18^ Treatment can have a significant deleterious impact in the patient's quality of life including issues with sexual function and faecal continence.^18^, ^19^, ^20^

Various studies have assessed HPV viral load (VL) in the different HPV‐driven cancers and its association with overall survival and prognosis and/or as a biomarker for lesion progression. In cervical cancers, low HPV viral load may implicate a worse prognosis (median value of 385.8 RLU/CO Kim et al. 2009^21^ 132.5 RLU/CO in Deng et al^22^). The number of copies of the HPV genome has also been examined in oropharyngeal cancer (OPC) cases driven by HPV and a number of studies have observed that high viral load is associated with a better prognosis versus low viral load.^23^, ^24^, ^25^, ^26^, ^27^ Additionally, disease recurrence has been shown to be significantly lower in those with high HPV load.^23^ For these publications median value ranged between 30.9, 132.5, 190 and 820 copies/cell.

Complimentary studies have investigated the implications of HPV viral load in and around the anal canal,^28^, ^29^, ^30^, ^31^, ^32^ however, the majority of the available publications have looked into HPV load in people living with HIV. Other studies have assessed VL in anal cancer^31^, ^32^ and found a median of 7.40 and 134 copies/cell. However, to our knowledge only one investigation has explored the association between HPV load, local control of cancer and overall survival.^31^ Authors found that patients with HPV16 DNA VL below the median viral load with low p16 expression showed significantly worse local control and overall survival (OS) than those with a VL above median.^32^

The reason behind why cases with HPV high viral load have better prognosis or OS is not completely understood, although it has been posited that it could be associated with the HPV episome status and integration as episome is associated with higher copies of HPV.^25^, ^33^, ^34^, ^35^

As is the case in most settings, there is no population‐based anal screening programme in place in Scotland (where the present work was performed) and generally, anal lesions are detected and managed, clinically. Moreover, as HPV testing is not currently included in the diagnostic work up of anal disease, there is no routinely collected information on HPV‐associated epidemiology in anal disease in Scotland. Proactive efforts are therefore needed to understand the nature and implications of HPV in anal cancer to inform prevention and management strategies of the future.

Our aim was to determine the HPV‐attributable fraction of anal cancers, that is, the fraction associated with HPV in Scotland, and to use this data to ascertain the influence of qualitative HPV status on clinical outcomes. Then, using this well annotated data set we also aimed to determine whether VL (in the HPV16 positive component) exerted an influence on clinical outcomes.

## 2 MATERIALS AND METHODS

### 2.1 Sample collation and timeframe

Squamous cell carcinoma of anus or anal canal samples (ICD‐11 coding 2C00.3) preserved in formalin fixed paraffin embedded (FFPE) blocks diagnosed between 2009 and 2018 were collated. These samples were originally obtained as part of standard of care for the management of patients with anal disease.^36^ All biopsies were obtained from the South‐east of Scotland representing 3 of 14 territorial health boards in Scotland; NHS Lothian, NHS Borders and NHS Fife. These health boards serve a population of 1,396,640 (data from 2019).^37^ Favourable ethical opinion to conduct the research was provided by University of St Andrews Teaching and Research Ethics Committee, reference MD 14482. This was further supported by approval for use of samples through the National Research for Scotland Bioresource (20/ES/0061), application reference SR1283.

Clinico‐demographic information was obtained on age, sex, stage of cancer (using the American Joint Committee on Cancer [AJCC] TNM system),^38^ response to treatment, date of diagnosis and vital (dead/alive) status. Age and stage of cancer were considered at the time of diagnosis. Information was obtained in January 2020 and indexed with a study number. Vital status information and date of death data were censored in July 2020. Response to treatment was aggregated in two different groups to simplify the analysis: response to treatment (including remission) and no response to treatment (including progression and recurrence). Cohort follow‐up started at date of diagnosis and continued until death or time of censoring.

Cases categorised according to the various clinical and demographic variables are summaried in Table 1. Age was stratified in four different groups: <50, 50–59, 60–69 and >=70. Response to treatment was organised in three groups: yes, no or unknown following the ESMO guidelines for anal cancer.^39^ Cancer stage was aggregated in five groups: I, II, III, IV and unknown following AJCC system effective January 2018.^38^

**TABLE 1:** Demographic & clinical characteristics of the anal cancer patients collected between 2009 and 2018 in the South‐East of Scotland. Stratification by HPV status and HPV16 Viral load

Variable	Level	HPV status & survival samples cohort *N* (%)			HPV16 Viral load & survival samples cohort *N* (%)			
		*n* = 185	HPV + ve (*n* = 164)	HPV‐ve (*n* = 21)	*n* = 145	Low (*n* = 47)	Medium (*n* = 50)	High (*n* = 48)
Sex	Female	120 (64.9%)	109 (66.5%)	11 (52.4%)	101 (69.7%)	29 (61.7%)	38 (76.0%)	34 (70.8%)
	Male	65 (35.1%)	55 (33.5%)	10 (47.6%)	44 (30.3%)	18 (38.3%)	12 (24.0%)	14 (29.2%)
Age	<50	28 (15.1%)	25 (15.2%)	3 (14.3%)	22 (15.2%)	7 (14.9%)	6 (12.0%)	9 (18.8%)
	50–59	48 (25.9%)	48 (29.3%)	0 (0%)	44 (30.3%)	18 (38.3%)	14 (28.0%)	12 (25.0%)
	60–69	56 (30.3%)	51 (31.1%)	5 (23.8%)	45 (31.0%)	17 (36.2%)	12 (24.0%)	16 (33.3%)
	70 and over	53 (28.6%)	40 (24.4%)	13 (61.9%)	34 (23.4%)	5 (10.6%)	18 (36.0%)	11 (22.9%)
Stage	I	27 (14.6%)	23 (14.0%)	4 (19.0%)	22 (15.2%)	5 (10.6%)	8 (16.0%)	9 (18.8%)
	II	68 (36.8%)	62 (37.8%)	6 (28.6%)	54 (37.2%)	15 (31.9%)	17 (34.0%)	22 (45.8%)
	III	53 (28.6%)	48 (29.3%)	5 (23.8%)	40 (27.6%)	15 (31.9%)	16 (32.0%)	11 (22.9%)
	IV	35 (18.9%)	23 (14.0%)	6 (28.6%)	25 (17.2%)	11 (23.4%)	9 (18.0%)	5 (10.4%)
	Unknown	2 (1.1%)	2 (1.2%)	0 (0.0%)	2 (1.4%)	1 (2.1%)	0 (0.0%)	1 (2.1%)
Response to Treatment	Yes	138 (74.6%)	125 (76.2%)	13 (61.9%)	111 (76.6%)	35 (74.5%)	37 (74.0%)	39 (81.2%)
	No	34 (18.4%)	28 (17.1%)	6 (28.6%)	23 (15.9%)	8 (17.0%)	9 (18.0%)	6 (12.5%)
	Unknown	13 (7.0%)	11 (6.7%)	2 (9.5%)	11 (7.6%)	4 (8.5%)	4 (8.0%)	3 (6.3%)
Vital status	Alive	124 (67.0%)	115 (70.1%)	9 (42.9%)	104 (71.7%)	29 (61.7%)	33 (66.0%)	42 (87.5%)
	Deceased	61 (33.0%)	49 (29.9%)	12 (57.1%)	41 (28.3%)	18 (38.3%)	17 (34.0%)	6 (12.5%)

*Note*: ‘N’s corresponds to the total number of samples for every category described above. Percentage (%) was calculated from the total number of valid samples (*n* = 185 & 145).

### 2.2 Overarching approach to HPV annotation: Qualitative analysis and quantitative analysis

A total of 185 anal cancer samples were annotated for HPV type‐specific prevalence, initially, using a PCR‐based assay: the Anyplex II 28 assay (Seegene, Korea) centrally at the Scottish HPV Reference Laboratory, Edinburgh, UK. One 10 μm section per sample was obtained and incubated in Seegene Univeral Lysis Buffer (LB) at 65°C overnight. DNA extraction was performed using the Microlab Nimbus IVD (Hamilton) with the StarMAg Universal cartridge Kit (Seegene). Mastermix was prepared with the Nimbus and PCR on the CFX Real‐time PCR instrument (Biorad).

This initial result allowed determination of type‐specific prevalence and examination of the association between qualitative HPV status (any HPV vs. no HPV) and survival, which we subsequently term the ‘qualitative analysis’. Given the dominance of HPV16 in the cohort, viral load analysis was restricted to samples that tested HPV16 positive, either as a mono infection or within a mixed infection. Once viral load was obtained (quantitative analysis), it was classified in three different groups: low, medium and high and linked to overall survival. A diagram illustrating the process followed can be found in Appendix A.

### 2.3 Measurement of viral load using ddPCR

Absolute quantification of viral load was performed on HPV16 + ve cancer samples (145 mono and nine mixed infections) using a droplet digital assay (ddPCR). This element of our analysis is referred to as the quantitative analysis group. Nucleic acid was extracted from HPV16 + ve samples (new section, 10 μm) using the QIAamp DNA Mini Kit (Qiagen) and sample concentration measurement was performed with the Qubit dsDNA High sensitivity kit (Thermo Fisher Scientific).

ddPCR was performed as described in Stevenson et al. 2020^23^ at the Centre for Virus Research, University of Glasgow. The RPP30 endogenous control probe primer set of 0.7 μl, HPV16 L1‐specific primers and probes at 300 nM (final concentration) respectively, 10–100 ng of template DNA and 1 μl of restriction digest mix (consisting of 4 U of both *Eco*RI and *Hind*III in 1x NEB Cutsmart buffer [NEB, UK]) were used for the mix. Reactions were mixed with Droplet Generation Oil on DG8 cartridges in the QX200 droplet generator (Bio‐Rad) to generate droplets. Thermal cycling conditions were: 95°C for 10 min followed by 40 × 30s at 94°C and 60°C for 1 min prior to final extension at 98°C for 10 min.

Post‐amplification, droplets were analysed on a QX200 Droplet Reader (Bio‐Rad), and output data files were analysed using QuantaSoft analysis software v1.7.4 (Bio‐Rad). The viral load for each sample was calculated relative to the endogenous RRP30 cellular gene internal control, with two copies present per cell. Any initially invalid results were repeated using a new FFPE section and fresh DNA extraction. After retesting, consistent invalids were not included in the analysis (*n* = 9).

### 2.4 Definition of viral load levels

The individual HPV16 viral loads were ranked from smallest to largest and separated using tertiles. The VL threshold(s) for L1 low viral load was <12.3, medium between 12.3 and 57 and high viral load above 57 copies/cell.

### 2.5 Association of HPV status and viral load with demographics and survival outcomes

Overall survival by qualitative status of HPV (HPV positive vs. negative; HPV16 positive vs. HPV negative) and HPV VL (low, medium and high) was analysed using the Kaplan–Meier method. The univariate and multivariate hazard ratios of HPV status (negative vs. positive) and virus load (low vs. medium and high) for all cause death were derived using Cox proportional hazard model. Two multivariate models were derived––age (<50, 50–59, 60–69, 70+) and sex adjusted model as described in Stevenson et al.^23^ and a fully adjusted model, where age, sex, stage (I, II, III, IV) and response to treatment (no, yes) were adjusted for. All the statistical analysis were performed using R‐studio (version 1.2.1335).^40^, ^41^, ^42^

For the qualitative detection HPV, the types 16, 18, 31, 33, 35, 39, 45, 51, 52, 56, 58, 59 and 68 were considered as high risk. HPV types considered as low‐risk HPV types include: 6, 11, 40, 42, 43, 44, 54 and 61.

## 3 RESULTS

### 3.1 Clinical/demographic characterisation of cohort

Overall HPV status stratified by demographic and clinical characteristics are presented in Table 1. The cohort contained 64.9% samples from females, and 35.1% males. The, majority of cases were diagnosed in individuals aged 60–69 (30.3%) and the majority of cases were stage II and III (36.7% and 28.6%). Additionally, 74.6% of cases responded to treatment and 67.0% were alive at date of censoring.

Of the female cases 90.8% were HPV positive; of the male cases, 84.6% were HPV positive. The majority of HPV positive cases were diagnosed in the 60–69 (31.1%) age range and were stage II (37.8%). Overall, 76.2% responded to treatment and 70.1% were alive at censoring.

With respect to the cases assessed for the quantitative analysis, 69.7% of samples were from females, 30.3% from males. The majority were diagnosed aged 60–69 years (31.0%), at stage II (37.2%). A total of 76.6% responded to treatment and 71.7% were alive at time of censoring. A higher proportion of those with high VL responded to treatment compared to those with low/medium VL (81.2% for the high VL group vs. 74.5% and 74.0% for the low and medium VL group). A higher proportion of those with high VL were alive at the time of censoring (87.5% for the high VL group vs. 61.7% and 66.0% for the low and medium VL group. (Table 1).

### 3.2 Qualitative analysis of HPV in anal cancer samples

A total of 185 anal cancer samples were genotyped for HPV. Of the 185 cases, 164 (88.6%) samples were positive for at least one HPV type. High‐risk (hr) types were detected in 87.03% of the samples. Monoinfection of HPV16 was present in 145 (78.4%) samples. Seven samples (3.8%) had a combination of HPV16 and other hr‐HPV type(s). Two samples (1.1%) were co infected with HPV16 and a low‐risk type. HPV18 was the second most dominant type, present in three samples (1.6%). HPV 33 and 68 were detected in two samples (1.1%), HPV 35, HPV 51 and HPV 52 in 1 (0.5%) while HPV 39 was detected in three (1.6%). The presence of low‐risk types without any other hr‐HPV was detected in three samples (1.6%).

### 3.3 Does HPV positivity have an impact on survival in patients with anal cancer?

Of the 185 cases included in the qualitative analysis, 61 (33.0%) patients died during follow‐up.

**FIGURE 1:**
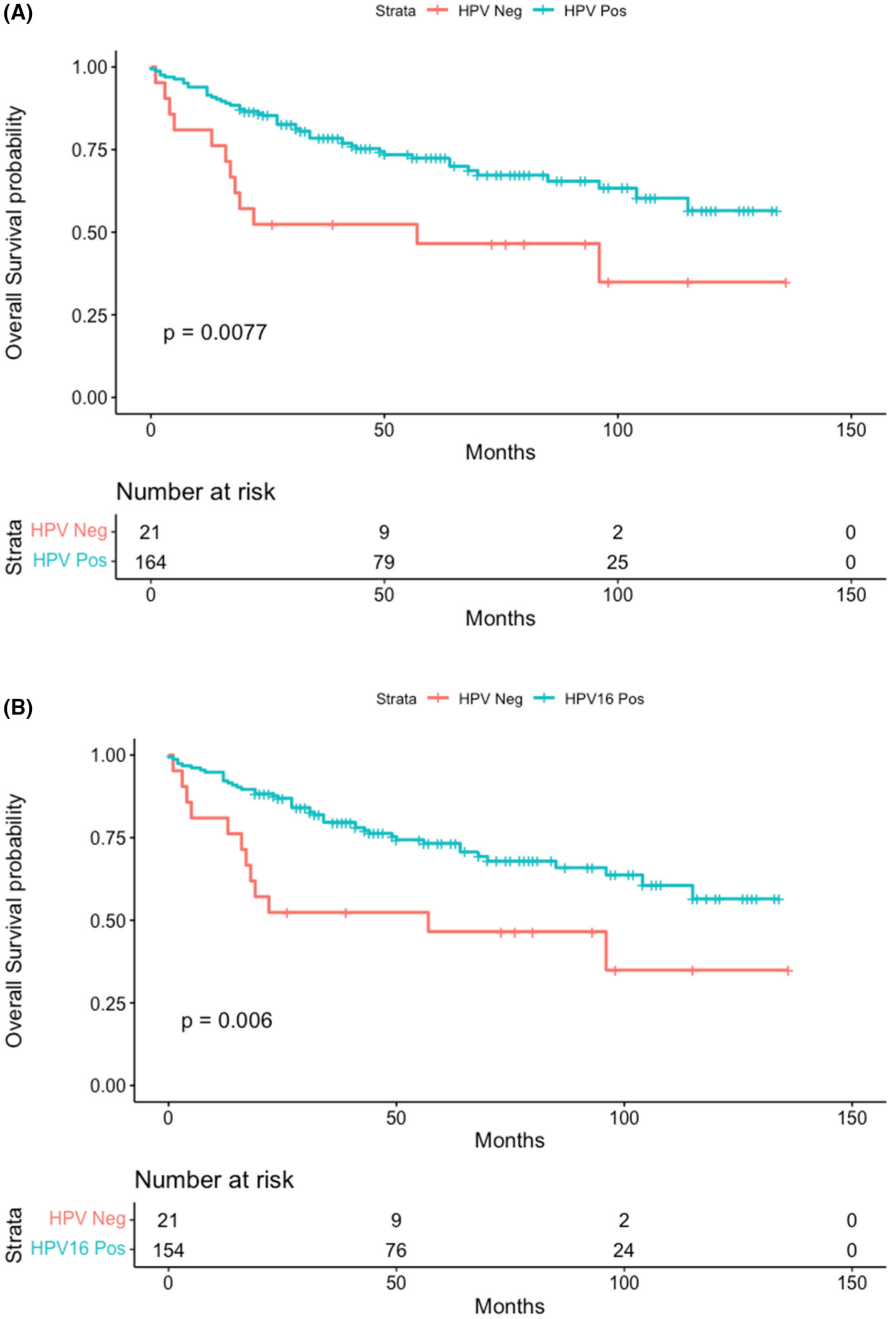
Overall Survival probability for ‘any’ HPV positive versus HPV negative cases (A) and for HPV16 positive and non‐HPV16 positive cases (B) using Kaplan–Meier estimator. Survival time is expressed in months from diagnosis date. Data censored in July 2020

Kaplan–Meier curves were produced and stratified by HPV status (positive and negative) (Figure 1A), and HPV16 status (Figure 1B). HPV positivity and HPV16 positive status were associated with better survival (log‐rank test *p* value 0.0077 and 0.006, respectively).

HPV + ve status was associated with improved overall survival in the univariate analysis with a hazard ratio (HR) of 0.44 (0.23–0.82, *p* = 0.01) (Table 2). In the univariate Cox model, variables associated with worse overall survival were Stage III; HR 5.0 (1.1–22), *p* = 0.003 and Stage IV HR 25.6 (6.0–109), *p* < 0.001 versus stage I and response to treatment 0.12 (0.07–0.33) *p* < 0.001 versus non response to treatment. After adjusting for age, gender, stage and response to treatment, HPV status continued to influence the overall survival, HR 0.24 (0.11–0.55) *p* < 0.001. When adjusting for age and gender alone, HR for HPV positive status was 0.41(0.21–0.82) *p* = 0.011.

**TABLE 2:** Univariate and multivariate hazard ratio of HPV status derived using Cox regression (*N* = 185)

Variable	Level	Unadjusted HR (95% Cis)	*p* value	Adjusted HR (95% Cis)	*p* value	Adjusted HR (95% Cis)	*p* value
HPV	HPV Neg	1		1		1	
	HPV Pos	0.44 (0.23–0.82)	0.01	0.24 (0.11–0.55)	<0.001	0.41 (0.21–0.82)	0.011
Sex	Male	1		1		1	
	Female	0.85 (0.51–1.4)	0.549	0.98 (0.52–1.87)	0.955	0.90 (0.53–1.53)	0.704
Age	<50	1				1	
	50–59	1.6 (0.68–3.7)	0.288	1.09 (0.43–2.79)	0.852	1.84 (0.77–4.37)	0.167
	60–69	1.2 (0.53–2.9)	0.635	2.40 (0.97–5.98)	0.059	1.31 (0.56–3.06)	0.532
	70 and over	1.6 (0.70–3.7)	0.257	1.88 (0.69–5.11)	0.217	1.48 (0.63–3.45)	0.365
Stage	I	1		1			
	II	3.5 (0.8–15)	0.095	4.28 (0.96–19.13)	0.057		
	III	5.0 (1.1–22)	0.003	5.67 (1.24–25.93)	0.025		
	IV	25.6 (6.0–109)	<0.001	18.58 (3.96–87.18)	<0.001		
Response to treatment	No	1		1			
	Yes	0.12 (0.07–0.21)	<0.001	0.16 (0.07–0.33)	<0.001		

### 3.4 Viral load range in the anal cancer samples

A total of 145/154 HPV16 positive samples (94.1%) were associated with valid reads in the ddPCR for HPV16 L1 sequences. Nine samples were excluded from the analysis because they generated less than 10,000 droplets (the threshold for validity) even after repeat testing.

Viral loads ranged from 0.021 to 710 copies of the HPV L1 gene per cell with a mean of 60.57 L1 copies. Those who were deceased at time of analysis (41/145, 28.28%) had a median L1 VL of 33.11 (IQR 3.6–43.5); while those still alive (104/145, 71.72%) had a median L1 VL of 74.57 (IQR 8.2–104.5) (Table 3). A total of 47 samples (32.4%) had a low VL, 50 a medium VL (34.5%) and 48 (33.1%) a high VL. Mean VL was 3.8, 35.6 and 148.9 for low, medium and high VL groups, respectively. Viral load stratified by vital status, irrespective of underlying demographics is described in Table 3.

**TABLE 3:** Viral loads obtained in the HPV16 + ve group

	Level	*N* (%)	Viral Load median
All	Low VL (<12.3)	47 (32.41%)	3.85
	Medium VL (12.3–57)	50 (34.48%)	35.65
	High VL (>57)	48 (33.10%)	148.93
	All	145	60.80 (IQR 5.74–85)
Alive	Low VL (<12.3)	29 (27.88%)	4.11
	Medium VL (12.3–57)	33 (31.73%)	37.16
	High VL (>57)	42 (40.38%)	152.61
	All	104	74.57 (IQR 8.25–104.5)
Deceased	Low VL (<12.3)	18 (43.9%)	3.43
	Medium VL (12.3–57)	17 (41.46%)	32.72
	High VL (>57)	6 (14.63%)	123.25
	All	41	33.11 (IQR 3.6–43.5)

### 3.5 Viral load and impact on clinical outcomes

Of those alive at the time of data censoring, 27.9% cancer samples were associated with a low VL, 31.7% a medium VL and 40.4% a high VL. Comparatively, in those who died low VL was present in 43.9%, medium VL in 41.5% whereas 14.6% had a high VL (Table 3).

**FIGURE 2:**
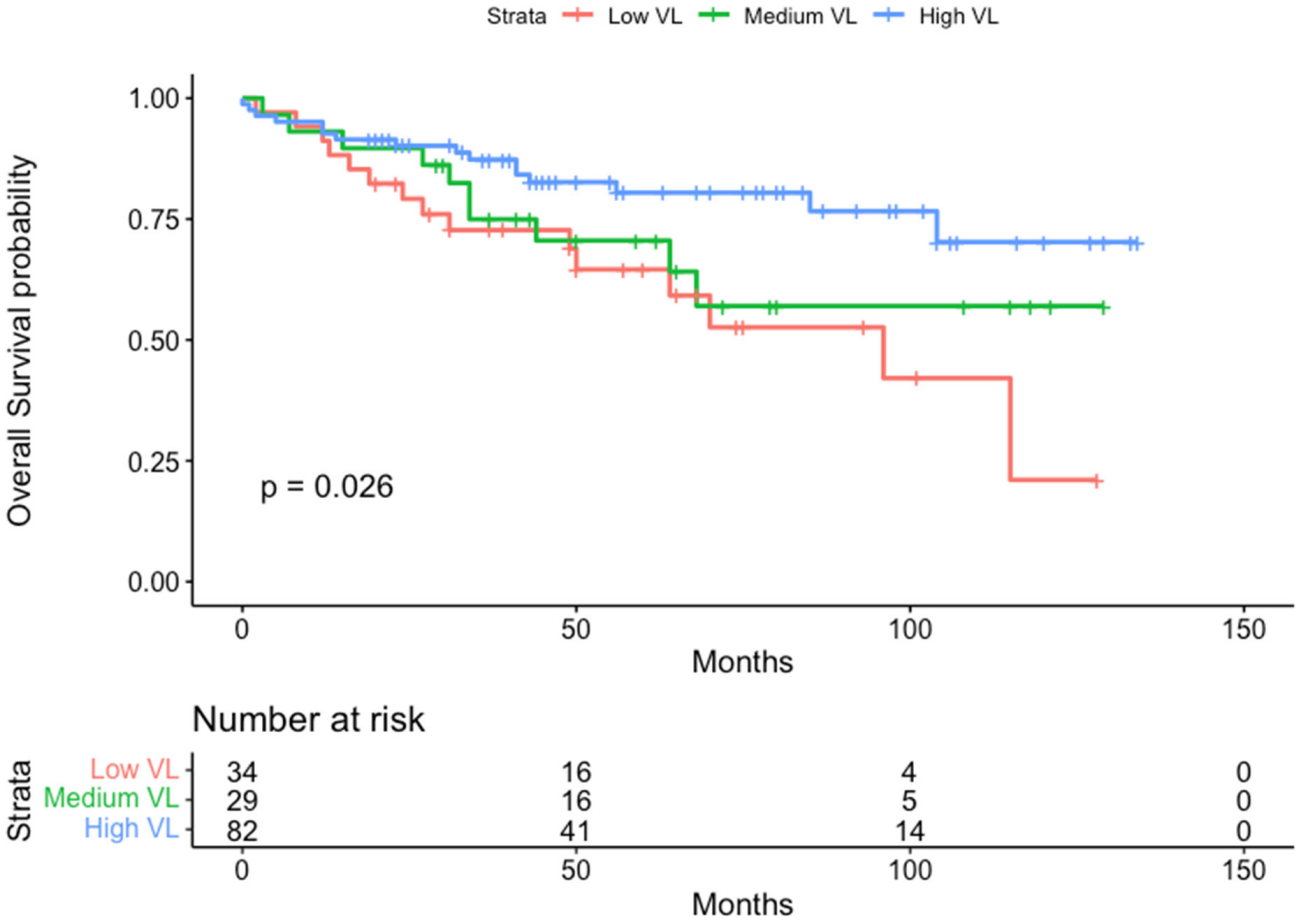
Kaplan–Meier survival curve stratified by viral load (Low, Medium and High). Survival time expressed in months from the diagnosis date. Data censored at 31st July 2020

For the Kaplan–Meier estimator, overall survival was calculated by classifying viral load in three groups, low, medium and high. Overall survival in those with medium and high viral load was higher than in those with low VL (*p* = 0.026), Figure 2.

Table 4 shows overall survival stratified by the clinical and demographic variables described in Table 1 with viral load categorised into the three tertiles with low VL as the reference. High viral load was associated with improved overall survival in the univariate analysis with a hazard ratio (HR) of 0.28 (0.11–0.71, *p* = 0.007) compared to low viral load. Variables associated with worse overall survival in the univariate model were Stage IV vs. stage I with HR of 25.2 (5.65–113), *p* < 0.001 and response to treatment [HR of 0.13 (0.064–0.27) *p* < 0.001] versus non response to treatment.

**TABLE 4:** Univariate and multivariate hazard ratio of L1 viral load derived using Cox regression (*N* = 145)

Variable	Level	Unadjusted HR (95% Cis)	*p* value	Adjusted HR (95% Cis)	*p* value	Adjusted HR (95% Cis)	*p* value
Viral Load	Low (<12.3)	1		1		1	
	Medium (12.3–57)	0.91 (0.47–1.76)	0.774	1.04 (0.45–2.40)	0.924	0.80 (0.40–1.60)	0.531
	High (>57)	0.28 (0.11–0.71)	0.007	0.39 (0.12–1.24)	0.111	0.27 (0.11–0.68)	0.006
Sex	Male	1		1		1	
	Female	1.2 (0.6–2.4)	0.625	1.09 (0.47–2.53)	0.838	1.35 (0.65–2.76)	0.419
Age	<50	1		1		1	
	50–59	1.51 (0.58–4.0)	0.398	0.77 (0.25–2.32)	0.639	1.30 (0.50–3.41)	0.588
	60–69	0.94 (0.34–2.6)	0.912	2.20 (0.69–7.01)	0.183	0.85 (0.30–2.40)	0.753
	70 and over	1.53 (0.56–4.2)	0.405	3.05 (0.757–12.31)	0117	1.65 (0.58–4.65)	0.347
Stage	I	1		1			
	II	2.2 (0.48–10)	0.302	2.31 (0.48–11.18)	0.299		
	III	2.9 (0.62–14)	0.178	2.58 (0.50–13.23)	0.254		
	IV	25.2 (5.65–113)	<0.001	21.52 (4.01–115.41)	<0.001		
Response to treatment	No	1		1			
	Yes	0.13 (0.064–0.27)	<0.001	0.23 (0.09–0.56)	0.001		

After adjustment for age, gender, stage and response to treatment, viral load did not significantly influence the overall survival; medium VL HR 1.04 (0.45–2.40) *p* = 0.924, high VL 0.39 (0.12–1.24) *p* = 0.111 compared to low VL. In the age/gender adjusted Cox model, high viral load was still associated with improved overall survival compared to low VL (0.27, 0.11–0.68) *p* = 0.006.

## 4 DISCUSSION

To our knowledge this is the first study looking at viral load in anal cancer samples and its association with overall survival. Most anal cancers included in this study were positive for HPV (88.6%), with HPV16 being the clear dominant type (93.3%) in the positive cases. This is consistent with the high positivity of HPV in anal cancer and the high prevalence of HPV16 reported by Desanjosé et al.^3^

We have identified that HPV status (HPV positive) was associated with improved overall survival in the univariate analysis compared to HPV negative cases. When adjusted, HPV status continued to influence overall survival. This aligns with the systematic review by Urbute et al., where the authors found HPV DNA positive anal cancers have significantly better OS compared with HPV negative cancers.^55^ Moreover, this observation is consistent with an emerging pattern in other cancers associated with HPV, including cervical,^43^, ^44^ oropharyngeal,^45^, ^46^ penile^47^, ^48^ and vulvar cancers.^49^

The ddPCR assay indicated that high viral load as measured by quantifying HPV16 L1 gene copies was associated with a better clinical outcome than low copies of L1 in the univariate analysis, when compared with low and medium VL. However, when Cox HR was adjusted, viral load did not influence the overall survival. This could be due to the relatively small sample size; as the confidence interval just exceeds one, it is possible that a larger study may tip into significance. If we consider the unadjusted analysis, our observations with viral load and survival are similar to those seen for oropharynx, where high viral load correlated with improved survival using the same ddPCR technology applied in the present study.^23^ Moreover, other investigators have shown a link with viral load and survival in cancers of the cervix,^21^, ^22^ head and neck^23^, ^24^, ^25^, ^26^, ^27^ and anus.^32^ Although use of viral load for active clinical management is still unclear; a biomarker of risk(s) that may inform multidisciplinary meeting discussion and/or serve as a marker for therapeutic strategies could have value.

The reason as to why higher viral load may plausibly confer a better prognosis (notwithstanding any association with stage) in HPV associated disease is not fully understood. It is feasible that a higher viral load in epithelial cells may maximise exposure to immune effector cells. Additionally, in cervical cancer cases, high viral load, mediated by integrated or episomal genomes has been shown to link with high levels of viral oncoprotein expression. For integrated genomes, epigenetic drivers and changes in stability of transcribed E6 E7 mRNAs are the main mechanisms of increased viral oncogene expression.^50^ By contrast, episomal genomes may have the ability to express the entire viral proteome and this may repress viral oncogene expression. For oropharyngeal cancers, studies have shown that those cases that have an episomal genome status and high viral load have a more favourable prognosis.^25^, ^33^, ^34^, ^35^ We did not explore physical status of the genome in the present work, but this would be an interesting area to explore in the future.

We decided to use ddPCR given the relative lack of data on the implications of VL in the anal disease context and the fact that ddPCR delivers a high precision,^23^, ^51^ arguably higher than that achievable by normalised real‐time PCR. Additionally, ddPCR platforms are likely to have an increasing role in service laboratories to support precision testing in solid and liquid biopsies including for the measurement of circulating HPV and tumour DNA.^52^, ^53^, ^54^

There are limitations to the study––although the sample set was well annotated it was still relatively small. A larger study would have conferred greater power to investigate the impact of viral load, particularly in view of the various adjustments. Also, we did not account for differences between margin versus canal tumours or percentage of tumour captured as this information was not available to us. Additionally, due to the relationship of HIV with anal cancer development, knowledge of HIV status and markers of immune status/competence would have been a valuable addition to our data set.

Notwithstanding the above limitations, we would argue that assessment of HPV status in anal cancer cases and viral load is worthy of further investigation. High viral load of HPV in anal cancer serves as a proxy for improved survival and may have potential as biomarker––particularly at an early stage in clinical management where response to treatment is unknown. Larger series and studies which investigate this relationship further are welcome.

## CONFLICT OF INTEREST

DG: Received gratis consumables from Seegene to support the HPV genotyping of the anal cancer samples of this study. KC: KC's institution has received research funding or gratis consumables to support research from the following commercial entities in the last 3 years: Cepheid, Euroimmun, GeneFirst, SelfScreen, Hiantis, Seegene, Roche, Abbott and Hologic. All other authors have nothing to declare.

## AUTHOR CONTRIBUTION

DG was involved in the lab testing, analysis of data, and drafted the manuscript. RG performed the data retrieval, and critical appraisal of the manuscript. AK and AS assisted with lab experiments and critical appraisal of the manuscript. JP assisted with the statistical analysis and critical appraisal of the manuscript. KK assisted with the statistical design. MTGH and SVG assisted in critical appraisal of the manuscript. KC was involved in planning, draft supervision and assisted in drafting and critical appraisal of the manuscript.

## ETHICS APPROVAL STATEMENT

Favourable ethical opinion to conduct the research was provided by University of St Andrews Teaching and Research Ethics Committee, reference MD 14482. This was further supported by approval for use of samples through the National Research for Scotland Bioresource (20/ES/0061), application reference SR1283.

## PATIENT CONSENT STATEMENT

Not applicable.

## Data Availability

Data in anonymized form can be made available upon reasonable request to the senior author, and following due process of governance and the Scottish Data Protection Regulations.

## APPENDIX A

### A.1

Summary of study flow used, including the qualitative identification of HPV in anal cancer and the viral load investigation confined to HPV16 + ve only samples.
